# Therapeutic strategies based on non-ionizing radiation to prevent venous neointimal hyperplasia: the relevance for stenosed arteriovenous fistula, and the role of vascular compliance

**DOI:** 10.3389/fcvm.2024.1356671

**Published:** 2024-02-05

**Authors:** Eliza Russu, Emil-Marian Arbanasi, Traian V. Chirila, Adrian V. Muresan

**Affiliations:** ^1^ Clinic of Vascular Surgery, Mures County Emergency Hospital, Targu Mures, Romania; ^2^ Department of Vascular Surgery, George Emil Palade University of Medicine, Pharmacy, Sciences and Technology of Targu Mures, Targu Mures, Romania; ^3^ Doctoral School of Medicine and Pharmacy, George Emil Palade University of Medicine, Pharmacy, Sciences and Technology of Targu Mures, Targu Mures, Romania; ^4^ Centre for Advanced Medical and Pharmaceutical Research (CCAMF), George Emil Palade University of Medicine, Pharmacy, Sciences and Technology of Targu Mures, Targu Mures, Romania; ^5^ Queensland Eye Institute, Woolloongabba, QLD, Australia; ^6^ Faculty of Medicine, George Emil Palade University of Medicine, Pharmacy, Sciences and Technology of Targu Mures, Targu Mures, Romania; ^7^ School of Chemistry and Physics, Queensland University of Technology, Brisbane, QLD, Australia; ^8^ Australian Institute of Bioengineering and Nanotechnology (AIBN), University of Queensland, St Lucia, QLD, Australia

**Keywords:** venous grafts, neointimal hyperplasia, arteriovenous fistula, non-ionizing radiotherapies, collagen crosslinking, vascular compliance

## Abstract

We have reviewed the development and current status of therapies based on exposure to non-ionizing radiation (with a photon energy less than 10 eV) aimed at suppressing the venous neointimal hyperplasia, and consequentially at avoiding stenosis in arteriovenous grafts. Due to the drawbacks associated with the medical use of ionizing radiation, prominently the radiation-induced cardiovascular disease, the availability of procedures using non-ionizing radiation is becoming a noteworthy objective for the current research. Further, the focus of the review was the use of such procedures for improving the vascular access function and assuring the clinical success of arteriovenous fistulae in hemodialysis patients. Following a brief discussion of the physical principles underlying radiotherapy, the current methods based on non-ionizing radiation, either in use or under development, were described in detail. There are currently five such techniques, including photodynamic therapy (PDT), far-infrared therapy, photochemical tissue passivation (PTP), Alucent vascular scaffolding, and adventitial photocrosslinking. The last three are contingent on the mechanical stiffening achievable by the exogenous photochemical crosslinking of tissular collagen, a process that leads to the decrease of venous compliance. As there are conflicting opinions on the role of compliance mismatch between arterial and venous conduits in a graft, this aspect was also considered in our review.

## Introduction

Carrel and Guthrie were the first to report the critical thickening of venous intima at anastomotic sites following the transplantation of veins into arteries (1). This process, now commonly referred to as *neointimal hyperplasia* (NIH), is recognized as a contributing factor to the stenosis events leading to complications and failures in interventions that involve venous conduits, such as coronary artery bypass grafting, coronary angioplasty, lower limb vein bypass, peripheral artery angioplasty, carotid endarterectomy, and arteriovenous fistula (AVF) or artificial graft for hemodialysis. The complex etiology and pathophysiology of NIH have been progressively unraveled through years of many physician-scientists' research, and excellently presented in several successive landmark reviews (2–14). In essence, the triggering event in the development of NIH is an injury to the vascular endothelium generated by turbulent vasculature hemodynamics. Following a series of other contributing events occurring over a period of post-anastomotic “arterialization”, the uncontrolled proliferation and migration of medial smooth muscle cells (SMCs) in tunica intima prevails and brings the hyperplasia process to a close, resulting in stenosis and loss of luminal patency. An abnormal arterialization is not compatible with a functional AVF, and the failure will occur rather sooner than later.

AVFs are crucial for maintaining the life and health of those afflicted by end-stage kidney disease and needing hemodialysis for survival. Failure of dialysis, leading to morbidity and mortality in such patients, is mainly caused by the vascular access dysfunction, which is a result of venous stenosis due to a complexity of pathophysiological events where NIH can play an aggravating role (15–18). The mechanism of the processes involved and their after effects on the vascular access, as well as the attempts to suppress them, are well documented (3, 4, 19–30).

The AVF, which was first introduced around 60 years ago (31, 32), has become the preferred form of vascular access for renal replacement therapies that involve dialysis (33, 34). Its functionality is traditionally assessed using the term “maturation”. According to some practical guidelines, a mature AVF must be able to deliver, ideally not later than 6 weeks after surgery, at least 300–600 ml/min blood for 3–5 h, and can be routinely cannulated with two needles. Although there is no consensus on an in-principle definition (19, 24), maturation can be reasonably qualified (32, 35, 36) as the ability of the inflow artery and the outflow vein to respond to the increased blood flow that occurs upon anastomosis of the two vessels.

Due to the low pressure in the venous system, immediately after performing AVF and exposing the venous wall to arterial pressure, the remodeling process is initiated through wall distension, endothelial injury, local ischemia, and cell apoptosis (37–39). Overtime, these will lead to generation of inflammatory process at the venous wall and NIH (38, 40). New methods, such as external stenting, have been developed and proposed to reduce NIH and local inflammation (41). However, none of these methods can be applied to AVF due to the requirement of periodic puncture of the venous component during dialysis sessions.

Amongst many therapeutic strategies against NIH-induced stenosis, the *ionizing* radiation has been employed due to its ability to kill cells, being administered for suppressing or reducing the proliferation of SMCs within the neointima. The method, which was pioneered (42) in hypercholesterolemic rabbit model, is classified as a sealed-source radiotherapeutic procedure, and it is known as endovascular (or intravascular) brachytherapy. Following animal experimentation, it has been applied episodically for treating stenosis associated with NIH in venous grafts including AVF, with variable outcomes (13, 24, 43–54). However, there is no clear evidence that the radiation therapies based suppressing the intimal SMCs had lasting benefits in preventing stenosis. Two forms of ionizing radiation were commonly used for the endovascular brachytherapy: particle radiation such as β radiation (beam of electrons), or electromagnetic radiation as *γ*-rays (photons). Radioisotopes of different half-lives were used as radiation sources, including ^192^Ir, ^32^P, ^48^V, ^90^Sr, or ^90^Y, and delivered in various formats such as seeds, pellets, tubes, wires, gels etc. In a different approach, the use of the ionizing region of ultraviolet (UV) radiation, UV-C, to inhibit SMC proliferation has been the subject of a U.S. patent (55), but its application has never been reported. We may conclude that no treatments based on ionizing radiation in human patients proved capable to assure the reverse of NIH in AVF.

The causal association between cardiovascular disease and exposure to ionizing radiation (either therapeutic, diagnostic or environmental), even at low dose, is of great concern (56–62), leading currently to revised principles for risk assessment and mitigation in patients undergoing radiotherapy, and to additional recommendations for cardiovascular management, protection and prevention (63, 64). This should be regarded as an argument for using the *non-ionizing* radiation as a substitute for the ionizing radiation, at least in vascular applications. The risk of radiation-induced cardiovascular disease remains present regardless of the location and type of therapeutic target.

A refreshment of the knowledge of the physics of non-ionizing radiation would be therefore beneficial. The radiation is energy emitted and transmitted as waves or particles through space or matter, and we are surrounded by electromagnetic, particle, acoustic, and gravitational radiations. Importantly, the first three types of radiation found applications in medicine. The radiations are commonly categorized into ionizing and non-ionizing, depending on the energy of the wave or particle, which ultimately determines the effect of irradiation onto the matter. While the particle radiations (α, β, protons, neutrons, positrons) are all of ionizing kind, the electromagnetic radiation presents a different situation. The current consensus holds that any radiation that carry an amount of energy higher than 10 eV is able to ionize atoms and molecules and cleave chemical bonds, therefore able to break down biomolecules. In the order of their increasing wavelength (i.e., decreasing energy), the electromagnetic spectrum contains the following regions: *γ*-rays, x-rays, ultraviolet, visible light, infrared, microwaves, and radio waves. The *γ*- and x-rays, with energies above 1,000 eV and much higher, can irreversibly damage cells and tissues. Ultraviolet (wavelength, 100–400 nm; photon energy, 12.4–3.1 eV) is the region where transition from ionizing to non-ionizing radiation occurs. As the 10-eV mark is situated within the ultraviolet-C region (100–280 nm; 12.4–4.43 eV), it appears that avoiding to irradiate biological tissue with UV-C would be a safe alternative. It is generally accepted that the UV-A region (315–400 nm; 3.94–3.1 eV) is biologically safe. Looking further, the photons in visible (400–700 nm; 3.1–1.7 eV) and infrared (700 –10^6^ nm; 1.8–1.24 × 10^–3^ eV) regions are not energetic enough to trigger ionization, therefore they are considered safe for most medical applications.

The aim of this review is to present the procedures based on non-ionizing radiations that have ever been employed to prevent NIH-induced stenosis, focusing on their application for AVF management. There are five such procedures in practice or development, and they are presented in Table 1. With the exception of a brief mention (21), the applications of non-ionizing radiation were rather ignored in all major reviews. We also discuss the role played in the NIH process by one of the fundamental mechanical characteristics of the blood vessels, the compliance (alternative terms include flexibility and pliability), which is the opposite of stiffness and can be defined as the ability of a body (in this case, the venous wall) to exhibit deformation upon the action of external forces.

**Table 1:** Summary of strategies based on non-ionizing radiation to suppress venous neointimal hyperplasia.

Method	Principle	Significance for AVF	References
Photodynamic therapy (PDT)	Irradiation with visible light to generate ROS that damage proliferating SMCs	Inhibition of NIH in animal models	a
Far-infrared therapy	Irradiation with FIR radiation^f^	# Human clinical trials # Improved AVF maturation # Increased vein diameter # Improved access flow # Improved AVF primary and secondary patency	b
Photochemical tissue passivation (PTP)	Irradiation with green light (max. at 550 nm) with rose Bengal to induce crosslinking of collagen and stiffening of venous wall possible cytotoxicity to SMCs	models including AVF	c
Alucent vascular scaffolding	Irradiation with visible light (400–525 nm) with naphthalimides to induce crosslinking of collagen and stiffening of venous wall	Promising outcome in animal AVF models	d
Adventitial crosslinking	Irradiation with UV-A rays (365 nm) with riboflavin to induce crosslinking of collagen and decrease venous compliance	Ex-vivo human veins become significantly stiffer	e

AVF, arteriovenous fistula; FIR, far infrared; NIH, neointimal hyperplasia; PDT, photodynamic therapy; PTP, photochemical tissue passivation; ROS, reactive oxygen species; SMC, smooth muscle cell; UV, ultraviolet.

^a^ Barton et al. (65); Burgher et al. (66); Jerjes et al. (67); Houthoofd et al. (68).

^b^ Lin et al. (69); Lin et al. (70); Bashar et al. (71); Wan et al. (72); Shemilt et al. (73); Lindhard et al. (74).

^c^ Goldstone et al. 75; Salinas et al. 76; Goldstone et al. 77; Goldstein et al. 78.

^d^ Shiu et al. (79); He et al. (80).

^e^ Arbanasi et al. (81).

^f^ Mechanism not fully elucidated.

### Photodynamic therapy

A photodynamic activity in biological systems involves photochemical reactions in which oxygen is consumed to generate bioactive harmful products, a process that generally elicits cell death, the target in this case being the SMCs. In the photodynamic therapy (PDT), this photochemical process is directed to the treatment of a disease and is based on the cooperation of three essential components: a photosensitizing agent, a radiation source with a specific wavelength, and tissular oxygen. Initially developed for treating cancers, PDT is currently used in several medical applications (82–84). In brief, the process commences with the absorption by the photosensitizer (PS) molecules, located within the target tissue, of the radiation provided by the source. Upon excitation due to irradiation, PS is transformed from its ground state (the singlet, ^1^PS) into a short-lived excited singlet state (^1^PS*), which within nanoseconds will dissipate its energy excess through three alternative routes: light emission (fluorescence), heat generation, or adoption of a more stable excited state (the triplet, ^3^PS*) through a process called intersystem crossing. The triplet has enough long lifetime (microseconds) to transfer its excess energy to the molecular oxygen (O_2_) in the tissue, generating singlet oxygen (^1^O_2_), which is short-lived and has a short radius of action, but is highly reactive and can induce oxidative damage and cell death. The triplet also has enough time to react directly with the tissular biomolecules and, through transfer of electrons or hydrogen extraction, generate free radicals, which by reacting with O_2_ produce reactive oxygen species (ROS), mainly the superoxide (O_2_^–**·**^) and hydroxyl (HO**^·^**) radicals, and hydrogen peroxide (H_2_O_2_). All ROS cause severe damage to cells.

The radiation commonly used for applying PDT is within a range from the end of visible (orange/red) to the beginning of near infrared regions, i.e., ∼600–850 nm, corresponding to photon energies between ∼2.1 and 1.46 eV. Sources include dye-pumped lasers, light-emitting diodes, or conventional lamps. The latter are generally avoided because unwanted thermal side effects. There is a large variety of photosensitizers used in PDT, and new agents are in continual development (82, 85, 86), as these compounds are key factors for a successful application of PDT. They must have radiation absorption peaks within the aforementioned wavelength range, and shall possess physiochemical characteristics that facilitate their optimal distribution and efficacy in the target tissues, as well as their elimination from the body. A photosensitizer from the class of porphyrins (Photofrin II) proved to be specifically cytotoxic *in vitro* to human atheromatous SMCs, even without photoactivation (87) Interestingly, the *in vitro* photodynamic activity of this particular photosensitizer was revealed by irradiating with UV-A rays the cultured SMCs, comparatively from non-atherosclerotic arteries and from stenosing lesions (88). Only 20% of the latter remained viable following irradiation. However, ultraviolet radiation is not used currently in PDT.

The efficacy of PDT in inhibiting arterial NIH was evaluated experimentally in animal models (rabbit, dog, rat) where the initial intimal injury was created with balloon catheters (89–91). The literature reporting the application of PDT for preventing NIH and for reducing formation of atherosclerotic plaques has been recently reviewed (68). The radiation levels used in these experiments were in the wavelength range of 600–710 nm, generated by lasers at fluences up to 100 J/cm^2^, in the presence of a variety of photosensitizers. The general conclusion was that PDT is a promising strategy against NIH. A study has been reported (67) in human patients with congenital vascular anomalies, who were exposed to 652-nm radiation provided by a diode laser at fluences of 10–20 J/cm^2^ in the presence of *m*-tetrahydroxyphenylchlorin as a photosensitizer. After an average follow-up of 21 months, 50% of the patients displayed good response to the PDT.

Related to the vascular access in hemodialysis patients, PDT was applied to a prosthetic arteriovenous graft (AVG), where the artery and vein were connected indirectly, through a tubular graft made of polytetrafluoroethylene (PTFE, Teflon). The study (65) was performed on dogs that had femoral AVGs implanted bilaterally. Four weeks after implantation, indium chloride methylpyropheophorbide (known as PhotoPoint™ MV6401) was administered as a photosensitizer to the animals. After creating injury in the veins with balloon catheters, the anastomotic sites were irradiated with light of 590 nm wavelength at a fluence of 100 J/cm^2^ (source not specified). Based on the thickness reduction revealed histologically, the authors concluded that PDT effectively inhibited the formation of NIH. The same group reported a similar study (66), but using the 664-nm radiation delivered by a diode laser and a photosensitizer known as MV2101 (composition not disclosed), also with similarly positive conclusions.

As far as our literature search has extended, there was no publication reporting the use of PDT for inhibition of NIH in non-prosthetic AVF, neither in animals nor in human subjects.

### Far-infrared therapy

In contrast to PDT, the use of far-infrared (FIR) radiation therapy for improving the blood flow, maturation and the patency of AVFs has been frequently reported, and was the objective of several human randomized clinical trials such as those presented in some major reviews (69–72, 74, 92, 93). It appears indeed that FIR therapy for AVF is a major application in cardiovascular medicine.

The non-ionizing far-infrared region is part of the infrared portion of electromagnetic radiation, which—in one of the many existing classifications—is contained between the wavelengths of 15 and 1,000 μm, corresponding to energies between 1.24 and 83 meV. Biologically, at such low energy, FIR is a safe radiation for medical applications. For vascular applications, emitters generating radiation within the wavelength range 3–25 μm (with a peak around 8 μm, energy 155 meV) are routinely employed. No sensitizing agents are required in this procedure. The trials reviewed in the above articles have involved thousands of hemodialysis patients (69–72, 74, 92, 93). It is important to mention that most of the FIR trials were not blinded. A range of evaluation criteria have been used, including changes in the access flow, survival number of AVF, quality of maturation, primary patency rate after one year of treatment, rate of stenosis and other AVF complications, cardiac output, assessment of inflammatory, vasoregulatory and endothelial functional factors, and changes in the content of asymmetric dimethylarginine (an inhibitor of nitric oxide synthase). However, based on changes in the content of vascular adhesion molecules, a recent clinical study (74) concluded that FIR might not have the expected vasoprotective effects, in spite of the previous favorable reports. In a prospective observational study including patients with both AVFs and AVGs (94), it was found that FIR therapy was not effective in preventing restenosis in AVFs after percutaneous translational angioplasty.

The mechanism of FIR therapeutic action is different from that of PDT and more complex. It may involve the reduction of growth rate of SMCs, but probably not by killing the cells. There are two effects of FIR, thermal and non-thermal, and they likely occur together. A number of mechanistic effects of FIR radiation have been revealed (69, 71, 73, 93, 95–97), such as vasodilation and increased access flow; angiogenesis; reduction of oxidative stress; release of anti-inflammatory factors; upregulation of endothelial nitric oxide synthase (eNOS); upregulation of heme oxygenase-1 (HO-1); inhibition of NIH. It is believed that all these effects may have a positive influence on the survival of AVFs.

### Photochemical tissue passivation

The technique of photochemical tissue passivation (PTP), sometimes referred to as photochemical tissue bonding (PTB), is based on the photochemical reactions occurring when tissues are irradiated with non-ionizing visible radiation in the presence of rose Bengal. This dye is a halogenated xanthene dianion that absorbs in the green region of the electromagnetic visible spectrum, with a maximum absorption peak at 550 nm. The photochemistry behind principles of PTP is identical to those underlying PDT, however the aim of PTP is to induce the crosslinking between tissular proteins (mostly native fibrillar collagen) leading to significant enhancement of tissues' mechanical properties (e.g., stiffening) and of their enzymolytic resistance, while the cells’ life is preserved.

The methodology for exogenous crosslinking of native collagen is related to the crosslinking of engineered collagen-based materials (98–100), and has led to several therapeutic strategies for treating disorders of the connective tissue. In contrast to many photosensitizing dyes, the rose Bengal molecule manifests a preference to bind strongly to tissular collagen (101, 102). The PTP technique has been developed at Wellman Center for Photomedicine (Harvard) in Boston (102, 103). An early vascular application was reported in animal (pig, rat) arteries (104), where it provided tight seals between anastomotic surfaces.

The direct inhibition of NIH was demonstrated (75) in porcine veins of which adventitia was subjected to 550-nm radiation emitted by a diode array light source at 90–120 J/cm^2^, in the presence of rose Bengal. Histologically, a decrease in SMC proliferation was also noticed following PTP, which may suggest accompanying free-radical-induced cytotoxicity, like in PDT. More important, due to photochemical crosslinking of adventitial collagen, the stiffness of veins increased tenfold. The scenario proposed (75) may explain the effect of PTP. When included into the arterial environment, a vein can be injured by mechanical pressure factors such as pulsatile stretching, turbulent flow, and shear stress in the wall. Exposed to the action of these factors, the vein becomes substantially distended, which triggers endothelial injury, followed by platelet aggregation, inflammatory processes, and specific signaling cascades, leading eventually to SMC proliferation, a key element in the formation of NIH. It was assumed that by strengthening the venous adventitia, the mechanical damage is significantly restricted. Similar results were reported by the same authors in *in vivo* studies in rat (76) and pig models (78). The role of venous compliance/stiffness is further discussed in the last section of this review.

We are aware of only one reported study regarding the efficacy of PTP in preventing NIH formation in AVF (77). PTP was performed on the vein prior to the creation of an AVF between the femoral artery and epigastric vein in rats, by irradiating with 550-nm light (diode array source) at a fluence of 25 J/cm^2^ and an irradiance of 87 mW/cm^2^. The animals were sacrificed and assessed one month after surgery. PTP reduced venous diametric dilation by ∼70% compared to controls; it also reduced ∼4 times the juxta-anastomotic intimal area, and ∼2.5 times total intimal area. An increase in the AVF flow was recorded following PTP, but not of high statistical significance. The investigators concluded that PTP might be considered as a promising therapy for preventing AVF failure.

### Alucent vascular scaffolding

This is yet another technique based on the same principle as PDT and PTP: irradiation with low-energy visible light in the presence of a photosensitizer. In fact, the only claim to the novelty of the procedure is that a new class of photosensitizers (substituted 4-amino-1,8-naphthalimides) was specifically developed for this purpose (105), although other chemical compounds were routinely available to fulfill that role. The current owner of the technology is Alucent Biomedical Inc. (Salt Lake City, UT, USA), who adopted an obvious misnomer for their proprietary product: “Natural Vascular Scaffold”. There is nothing “natural” in the concept, as the photosensitizer is a substance fully synthesized in laboratory, the radiation (wavelength 400–525 nm) is generated by manufactured instruments (xenon lamps or lasers), and luminal pre-dilation (which is crucial to the main application of the procedure) is achieved by standard balloon angioplasty. The procedure was initially intended to replace percutaneous transluminal angioplasty/stenting as a treatment for peripheral artery disease (106). It was claimed (105) that because only a limited number of crosslinks are produced in collagen, the resulting stent-like constructs will be not as rigid as the stents in current use. However, no explanation has been offered for a strategy that would be able to “limit” the extent of a photochemical crosslinking process.

The technique was applied to AVF in animal models. In such a study (79), an AVF was created between femoral vein and femoral artery in rats. After restoring blood flow, a solution of the photosensitizer (4-amino-1,8-naphthalimide) was dropped on the anastomotic site, and after 5 min the area was irradiated with 450-nm light (source not specified) for 1 min. Animals were sacrificed one month later, and tissue specimens were harvested and processed to be analyzed by histology, morphometry, immunohistochemistry, and microscopy. It was found that in the treated vessels the luminal area was larger than in controls, while the contents in IL-6 and MMP markers were significantly reduced. It was also surmised that the changes detected in the structure of native collagen might have a beneficial effect on AVF maturation. In a recent *in vivo* study (80), the method was applied to sheep cephalic veins using a balloon catheter coated with the photosensitizer and carrying a light fiber through which 450-nm radiation was delivered. The resulting luminal area was larger in the treated animals, where an increased number of SMCs was also observed, without noticeable NIH. In the same study, donor human saphenous and cephalic veins were subjected to the same treatment, and then to a distensibility assessment. The treated veins could tolerate up to 66% overstretch.

### Adventitial photocrosslinking

Mechanical augmentation of aortic adventitia by irradiating it with UV-A rays in the presence of riboflavin has been demonstrated in *ex-vivo* porcine abdominal aortas (107), and proposed as a method to lower the risk of rupture of abdominal aortic aneurysms. The reinforcement effect is due to the riboflavin-photosensitized crosslinking of the adventitial collagen, and was shown to take place even if the adventitial specimens were experimentally degraded by collagenolysis (108) or elastolysis (109). There are no doubts about the chemistry underlying the method, as the radiation-induced, riboflavin-photosensitized crosslinking of tissue proteins is based on well elucidated and understood photochemical processes (102).

The method has been recently extended to the venous wall (81), with an aim at reinforcing it mechanically as a potential method to inhibit the development of NIH, of obvious relevance to the vascular access through AVFs. Whole-thickness wall specimens of human superficial femoral vein and great saphenous vein (GSV), retrieved form a patient who underwent limb amputation, were soaked in riboflavin solution and then exposed to UV-A radiation (365 nm) for 3 min at an irradiance of 50 mW/cm^2^. The samples were evaluated biaxially in a specialized biomechanical tester, before and after the radiative treatment. The measured Young's modulus (representing stiffness—the reciprocal of compliance) of the GSV specimens increased significantly after irradiation, by ∼120% longitudinally and ∼80% circumferentially, proving the efficacy of the method. In addition, specimens of superficial femoral artery were also included in the study (81), and the investigators found that the mechanical behavior of the irradiated vein became similar to that of the non-irradiated artery. In these experiments, the adventitia was not separated from the wall. As it is unlikely that the radiation penetrated further into the tunica media, the adventitia took up the whole radiation output. However, the mechanical effect was global, reflected in enhanced stiffness and strength of the entire venous wall. An additional complication warranting consideration of UV-A exposure as a novel therapeutic approach is the development of aneurysms in AVF, in the case where during surgery we registered an important increase in vein diameter. In instances where surgical intervention is lacking, the progression of these aneurysms may culminate in rupture, precipitating hemorrhagic shock and eventual fatality (110, 111).

### Role of venous compliance

The compliance of the venous wall and the pre-operative diameter of the vein play an important role in AVF dysfunction. Numerous studies have tried to identify an optimal pre-operative venous diameter threshold to ensure AVF maturation (111–113). Thus, according to the guidelines of the European Society of Vascular and Endovascular Surgery, a minimum internal diameter for the arterial and venous component of 2 mm in the case of radiocephalic AVF (RCAVF) and a minimum of 3 mm in the case of brachiocephalic AVF (BCAVF) and brachiobasilic AVF (BBAVF) is recommended (113). Similarly Kaller et al. (111), demonstrated that a diameter greater than 2.25 mm for the radial artery and 2.55 mm for the cephalic vein is associated with a higher maturation rate in the case of RCAVF. Recently, another study (112) identified that a pre-operative diameter smaller than 2.95 mm for the artery and 2.15 mm for the vein is associated with AVF dysfunction. Moreover, other studies (114–116) have demonstrated the importance of increasing the venous diameter (intra-operatively and immediately post-operatively) in the favorable evolution of AVF. In contrast, there are several published studies (117–122) showing that by reinforcing the vein graft via an external stent/sheath made of synthetic polymers, the stiffness was enhanced, lessening the compliance mismatch, and resulting in the reduction of SMC proliferation, thus inhibiting hyperplasia.

It can be seen from the above overview (and also shown in Table 1) that three of the methods developed to inhibit NIH in venous grafts are relying on the mechanical reinforcement of the venous wall induced by photochemical crosslinking of tissular collagen, while exposed either to visible radiation, like in PTP (75–78, 104) or Alucent technique (79, 80), or to UV-A radiation, like in adventitial crosslinking method (81). The underlying hypothesis was that the reduction of vascular compliance in the venous component (equivalent to the increase of its stiffness), intended to also reduce the compliance mismatch between the two different conduits, lowers the risk of endothelial injury induced by an abnormal distension of the vein following its grafting to an artery. The role of hemodynamics in the progression of NIH and in vein-grafting outcomes has been intensively investigated over the past three decades (123, 124). It was suggested (123) that no less than 9 different mechanical effects can act upon a grafted vein conduit after being exposed to arterial pressure and flow. On the other hand, it was found (125–129) that following experimental mechanical stretching of venous conduits *in vitro* or in animal models, the growth factors promoting SMC proliferation were upregulated. Can an increase, achieved by exogenous means, of the venous conduit's stiffness neutralize such effects? Moreover, what shall be done about the compliance of the arterial conduit? These issues are complex and encumbered by dissenting findings or hypotheses.

Regarding the arterial component in the AVF, a review (130) showed that a stiffer arterial conduit contributes to the failure of AVF maturation, extending the conclusion to veins too with no valid reason, and recommending a general reduction of stiffness as a preventive treatment. However, other studies (131–135) failed to establish a definite role of arterial stiffness in the maturation of AVF.

Regarding the compliance of venous conduits in AVFs, two studies on human patients that included biomechanical evaluation either by plethysmography (136) or by dynamic mechanical analysis (137) have demonstrated higher failure rates with reduced venous compliance (i.e., with increased stiffness). It has also been reported that venous compliance decreases naturally with age (138) and that in hypertensive hemodialysis patients, the compliance is reduced irreversibly (139). These findings seem to cast doubt on the possibility of preventing NIH by reducing venous compliance, achievable through the cross-linking of vascular collagen. However, this issue is far from being resolved because the results of the animal model contradict (41, 140) the above-mentioned information. This obviously suggests that more research on this subject would be both relevant and beneficial. So far, FIR therapy has shown to be highly advantageous for patients with AVF, where there is no notable increase in venous diameter immediately after surgery. This therapy effectively increases the maturation and long-term patency of the AVF. As for other therapeutic strategies, while published results are promising, further studies are needed to establish the criteria for their applicability.

## Conclusions

In an attempt to counter the disadvantages of therapies based on ionizing radiation, such as the risk of induced cardiovascular disease and increasingly stringent safety requirements, procedures using non-ionizing radiation are currently developed and assessed as means to inhibit neointimal hyperplasia and prevent stenosis in venous grafts. This is of crucial importance in the quest for reducing the failure rate of arteriovenous fistula in hemodialysis patients.
